# Hypovirulence of *Sclerotium rolfsii* Caused by Associated RNA Mycovirus

**DOI:** 10.3389/fmicb.2016.01798

**Published:** 2016-11-10

**Authors:** Jie Zhong, Dan Chen, Hong J. Zhu, Bi D. Gao, Qian Zhou

**Affiliations:** Hunan Provincial Key Laboratory for Biology and Control of Plant Diseases and Insect Pests, Hunan Agricultural UniversityChangsha, China

**Keywords:** Mycovirus, hypovirulence, *Sclerotium rolfsii*, hypovirus, dsRNA

## Abstract

Mycoviruses associated with hypovirulence are potential biological control agents and could be useful to study the pathogenesis of fungal host pathogens. *Sclerotium rolfsii*, a pathogenic fungus, causes southern blight in a wide variety of crops. In this study, we isolated a series of dsRNAs from a debilitated *S. rolfsii* strain, BLH-1, which had pronounced phenotypic aberrations including reduced pathogenicity, mycelial growth and deficient sclerotia production. Virus-curing and horizontal transmission experiments that eliminated or transmitted, respectively, all dsRNA elements showed that the dsRNAs were involved in the hypovirulent traits of BLH-1. Ultrastructure examination also showed hyphae fracture and cytoplasm or organelle degeneration in BLH-1 hyphal cells compared to the virus-free strain. Three assembled cDNA contigs generated from the cDNA library cloned from the purified dsRNA indicated that strain BLH-1 was infected by at least three novel mycoviruses. One has similarity to the hypovirulence-associated Sclerotinia sclerotiorum hypovirus 2 (SsHV2) in the family *Hypoviridae*, and the other two are related to two different unclassified dsRNA mycovirus families. To our knowledge, this is the first report of *S. rolfsii* hypovirulence that was correlated with its associated dsRNA.

## Introduction

Mycoviruses (also denoted as fungal viruses) are widely described in major fungal groups, including most of the plant-pathogenic fungi (Ghabrial and Suzuki, 2009; Pearson et al., 2009; Xie and Jiang, 2014). All mycoviruses reported to date consist of double-stranded RNA (dsRNA), positive-sense single-stranded RNA (+ssRNA), the sole examples of negative-sense single-stranded RNA (-ssRNA), and single-stranded DNA (ssDNA) (Yu et al., 2010; Liu et al., 2014; Ghabrial et al., 2015). Generally, many mycoviruses are associated with latent infections in their fungal hosts. However, some mycoviruses can induce phenotypic aberrations or alterations, including hypovirulence and debilitation in their phytopathogenic fungal hosts. These mycoviruses are valuable for exploitation as biological agents to combat fungal diseases. In Europe, hypovirulent isolates of *Cryphonectria parasitica* infected by Cryphonectria hypovirus 1 (CHV1) have successfully been used to control chestnut blight caused by virulent strains of this pathogen (Nuss, 2005). Moreover, a hypovirulence-associated DNA virus, SsHADV1, also showed the ability to control Sclerotinia disease under field conditions (Yu et al., 2010, 2013), similar to Rosellinia necatrix megabirnavirus 1 (RnMBV1), which showed significant potential for the biological control of apple white root rot disease (Chiba et al., 2009). Due to their potential use in biological control, studying the phenomenon of mycovirus-mediated hypovirulence is attractive for numerous researchers. In addition, the hypovirulence-associated mycoviruses, which have divergent molecular characteristics, will also enhance our understanding of virus diversity and evolution and contribute to the establishment of host-mycovirus systems (i.e., Cryphonectria parasitica-hypovirus, Helmintosporium victoriae-HvV190S, Sclerotinia sclerotiorum-mycovirus, Rosellinia necatrix-mycovirus, and Fusarium graminearum- mycovirus), which are beneficial for studying virus-host interactions including fungal and viral pathogenesis at the molecular level (Xie and Jiang, 2014; Wang et al., 2015).

*Sclerotium rolfsii* Sacc. [*Athelia rolfsii* (Curzi) Tu Kimbrough], a soil-borne fungus with a broad host range of more than 600 species (Rivard et al., 2010), is the causal agent of southern blight disease in a wide variety of crops. It forms brownish sclerotia that can survive in soil for long periods, making this disease difficult to control (Elad, 1995). Currently, the control of this serious plant disease is mainly based on the application of chemical pesticides; however, this strategy may give rise to fungicide resistance and environmental pollution. Consequently, screening for alternative environmentally friendly disease control measures, such as the use of mycoviruses possessing biological control potential, is attractive and significant. However, to date, no mycovirus has been reported for *S. rolfsii*.

In 2014, we isolated *S. rolfsii* strain BLH-1 from *Macleaya cordata*. When cultured on PDA and compared to other *S. rolfsii* strains isolated from other host plants, abnormal phenotypic traits in BLH-1, including slow growth, deficiency in sclerotia production and attenuation in pathogenicity, were occasionally found. In many cases, fungal phenotypic aberrations or alterations are attributed to mycovirus infection, by which we may explore novel biological control agents to combat the relevant plant pathogenic fungi. In addition, attenuated strains with abnormal phenotypic traits would be useful to study the pathogenesis of plant pathogens. In this study, we carried out experiments to (i) determine whether dsRNA mycoviruses were present in the attenuated *S. rolfsii* strain BLH-1, (ii) to determine the relationship between the dsRNAs and the discernible phenotypic changes of *S. rolfsii*, and (iii) to identify the molecular characteristics of partial dsRNA.

## Materials and Methods

### Fungal Isolates and Culture Conditions

The *S. rolfsii* strain BLH-1 analyzed in this study was collected from a diseased *M. cordata* plant in the Hunan province of China and shared a series of dsRNA elements ranging from 1 to 15 kbp in size. Its identity was determined by rDNA-ITS sequencing, with the accession number KU885934. Derivative dsRNA-free isolates, represented by BLH-1-T1 and BLH-1-P1, were obtained from the paternal strain BLH-1 in hyphal tip culture and protoplast regeneration experiments, respectively. *S. rolfsii* strain LJ-01 was isolated from the roots of pepper plants infected by southern blight disease. All isolates and strains were cultured on potato dextrose agar (PDA) at 27°C.

### Curing of dsRNA

#### Protoplast Regeneration

Spheroplasts were prepared by the method described in Moleleki et al. (2003) and Kanematsu et al. (2004), with minor modifications. Briefly, mycelial plugs from the actively growing PDA plate were transferred to potato dextrose (PD) broth with shaking (170 rpm) at 28°C for 7 days and then transferred to fresh PD with shaking culture for another 24 h. Mycelia were collected by filtering through four layers of gauze and washed once with osmoticum (0.7 M MgSO4). The mycelia were resuspended in a filter-sterilized enzyme-osmoticum mixture (containing 1% Snailase [Sigma], 1% Driselase [Sigma] and 0.1% Lysing Enzymes [Sigma]) and incubated at 28°C with gentle shaking for 4 h. Then, the cultures were filtered through a 120-μm pore size nylon mesh to remove debris. Protoplasts were precipitated by centrifugation at 3,000 × *g* for 10 min, washed twice with osmoticum, and resuspended in an appropriate volume of STC (1 M sorbitol, 50 mM Tris HCl [pH 8], 50 mM CaCl2). Final concentrations of the protoplast suspension were determined with a hemocytometer and maintained on ice. Finally, portions (200 μl) of the spheroplast preparation were gently mixed with 20 ml dissolved regeneration medium (1 g of casein hydrolysate per liter, 1 g of yeast extract per liter, 342 g of sucrose per liter, 16 g of agar per liter) in petri dishes and incubated at 27°C in the dark for 1–2 days. Blocks of agar from the edges of each single colony were collected and placed in fresh PDA plates.

#### Hyphal Tip Isolation

Firstly, mycelial plugs were grown on PDA plates at 27°C for 2 days in the dark. Then, tips from single hyphae were removed from the colony margin with the aid of a dissecting microscope and transferred onto fresh PDA for four more cycles of hyphal tip isolation and culture. All regenerated isolates were randomly transferred to PD broth culture for mycelial collection and subjected to dsRNA extraction to detect mycovirus infection.

### Horizontal Transmission of Hypovirulence and the Associated dsRNAs

To determine if the hypovirulent trait of *S. rolfsii* BLH-1 was associated with dsRNA elements and was transmissible through hyphal anastomosis, pairing culture experiments for horizontal transmission were conducted (Wu et al., 2007, 2012). The dsRNA-containing hypovirulent strain BLH-1 was used as the donor, and virus-free virulent strains BLH-1-P1 and LJ-01 were recipients. Mycelial plugs of the two donor and recipient combinations (BLH-1/BLH-1-P1; BLH-1/LJ-01) were individually cultured 1 cm apart on a PDA dish. Mycelial agar plugs were taken from the edge of each colony of the two recipients (BLH-1-P1 and LJ-01) to obtain recipient derivative isolates.

To test if the dsRNAs could be transmitted horizontally to other fungal species, *Botrytis cinerea* strain HM-03 and *Sclerotinia sclerotiorum* strain JH-05 were individually cultured using the pairing culture technique with BLH-1 on a PDA plate as described above. Each of the recipient derivative isolates was incubated in PDA and subjected to detection for the presence of dsRNA.

### Pathogenicity Assay

Pathogenicity tests were conducted on potato (*Solanum tuberosum*) and pepper (*Capsicum annuum*) to evaluate the virulence of the *S. rolfsii* strains. The stems of these seedling plants were needle-pricked in the center near the root and covered by mycelial plugs (0.5 mm in diameter) that were removed from the colony margin of PDA cultures of each strain. Control seedlings were mock-inoculated with sterile PDA plugs without mycelia growing in them. Alternatively, the plants were inoculated by diapiric injection of the stems near the root using toothpicks covered with mycelium produced by co-culture of autoclave-sterilized toothpicks and fungal strains on PDA plates. In the same way, diapiric injections with only autoclave-sterilized toothpicks were conducted as controls. All inoculations were individually sealed by wrapping them with absorbent cotton and kept in a greenhouse at 27°C and 80% relative humidity. Lesion length on each inoculated plant was measured after the disease symptoms developed 5–8 days post-inoculation. These tests were repeated once, with each having three replications. The fungal strains were re-isolated, and the presence of dsRNA was checked by dsRNA extraction and agar gel electrophoresis as described above.

### Comparisons of Mycelial Growth and Colony Morphology

Colony morphology and growth rates of the original virus-infected strain BLH-1 and the putatively virus-free derivative isolate BLH-1-P1 were compared by growing on PDA plates under the same culture conditions at 27°C. For these comparisons, mycelial agar plugs were picked from the colony margins of 2-day-old PDA cultures of each strain and transferred to the center of petri dishes containing 15 ml PDA. The colony diameter of each dish was measured daily, from 1 day after incubation until the mycelium had covered the entire plate. The colony morphology and formation of sclerotia were examined at approximately 5 and 14 days, respectively. The formula RGR (cm/day) = (D3 - D2)/2 was used to calculate the mycelial growth rate, where D2 and D3 represent the diameters of 2- and 3-day-old colonies in each dish, respectively. These tests were repeated once with each strain cultured for five dishes (replicates).

### Enzyme Activity Assays

Assays for laccase activity were conducted by adding 0.01% 2,2-Azino-bis 3-ethylbenzthiazoline-6-sulfonic acid (ABTS) to the PDA plate (Guetsky et al., 2005). Laccase activity was indicated by formation of green color after mycelial agar plugs had been transferred to the agar plate. Laccase activity was also assayed using mycelial culture filtrate dropped upon holes in an agar plate containing 0.01% ABTS. The culture filtrates were prepared by culturing the fungal strains in PDB (50 ml) on a shaker (180 rpm) at 27°C for 5 days and then filtered through a filter paper. The laccase activity was also indicated by formation of green color around the holes in agar plate where culture filtrates were dropped.

Colony surface hydrophobicity was tested using bromophenol blue as described by Yu J. et al. (2015). A 15-μl droplet of water containing bromophenol blue was added to fungal colonies of dsRNA-free and dsRNA-infected strains cultured on PDA agar.

For cellulase activity assays, mycelial agar plugs were cultured on PDA containing 20 g/l carboxymethyl cellulose sodium salt for 2 days and then stained with Congo red (Liao et al., 2012). We regard the diameter of a bright yellow color as an indicator of cellulase activity.

Acid-producing ability was tested by placing mycelial agar plugs on PDA containing 0.01% (wt/vol) bromophenol blue for 5 days (Xiao et al., 2014). The appearance of yellow in the culture medium was regarded as an indicator of acid production.

Quantitatively assay of the oxalic acid production was performed using the method described previously. Mycelial agar plugs of the two fungal strains were inoculated in PD with shaking at 150 rpm at 27°C for 5 days. Culture filtrates were retrieved from three flasks of each strain and subjected to pH assesse. The concentration of oxalic acid was determined using high-performance liquid chromatography (Xiao et al., 2014).

### Scanning Electron Microscopy (SEM) and Transmission Electron Microscopy (TEM)

Scanning electron microscopy was used for hyphal surface structure examination of the hypovirulent strain BLH-1 and virus-free virulent strain BLH-1-P1. The mycelia used for ultrathin sections of the two strains were collected from the 5-day cultured PDA petri dish. Samples were dehydrated with a graded series of acetone (30, 50, 70, 80, 90, and 100%; vol/vol), mounted on stubs, sputter coated as previously described (Li Y. et al., 2011), and then examined with a scanning electron microscope.

Subcellular characteristics of the two strains were also examined by TEM. Mycelia were collected from a sterilized coverslip inserted in a PDA petri dish and cultured with fungal strains for 5 days. The mycelial specimens were fixed and dehydrated using conventional procedures described previously (Wu et al., 2007). Ultrathin sections (50–60 nm in thickness) for the mycelial specimens were cut using a diamond knife, mounted on slotted, formvar-coated grids and stained with 5% aqueous lead citrate and 5% uranyl acetate. Finally, the ultrathin sections were examined under TEM.

### Extraction of dsRNA

The presence of dsRNA molecules can represent the genomes of dsRNA mycoviruses or the replicative forms of single-stranded RNA (ssRNA) mycoviruses. Extraction of dsRNAs from mycelia of each fungal strain was conducted using the cellulose chromatography method described by Morris and Dodds (1979). Mycelial mass was collected from PD broth culture in an orbital shaker at 110 rpm for 4 to 7 days at 27°C and by filtration through four layers of cheesecloth, followed by washing with distilled water. Then, the dsRNAs were extracted and verified when sequentially treated with RNase-free DNaseI and S1 nuclease at 37°C for 30 min. The dsRNA size was estimated by 1% agarose electrophoresis and staining with 0.5 μg/ml ethidium bromide under UV.

### cDNA Synthesis, Cloning, and Sequence Analyses

We used cDNA library synthesis to partially characterize the dsRNAs from the hypovirulent strain BLH-1 based on the method described previously (Zhang et al., 2014). DsRNAs were purified from electrophoresed agarose gel and then served as a template to generate cDNA fragments with random hexanucleotide primers and reverse transcriptase. After the initial round of sequencing, sequence gaps that were not covered were filled by RT-PCR amplification using designed primers based on the obtained cDNA sequences flanking the gaps. All amplified cDNA products were cloned into the pMD18-T vector (TaKaRa) and sequenced at least three independent times for every base. Homology searches were conducted using the BLAST program against the National Center for Biotechnology Information (NCBI) databases^1^. Multiple sequence alignments were carried out using the ClustalX program (Larkin et al., 2007). A phylogenetic tree was made based on sequence alignment using the neighbor-joining (NJ) method, with a bootstrap test of 1,000 re-samplings in MEGA 6 (Tamura et al., 2013).

## Results

### Strain BLH-1, Which Lost the Capacity to Produce Sclerotia, Carries dsRNA Elements

**FIGURE 1:**
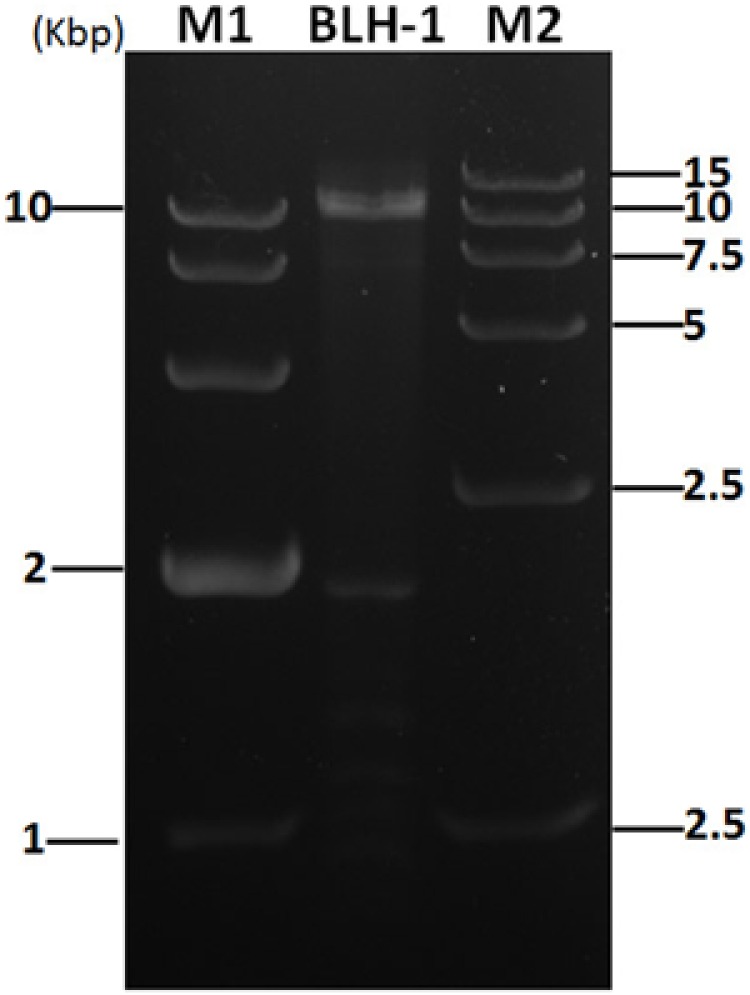
**dsRNA segments extracted from strain BLH-1 using the cellulose chromatography method were agarose gel-electrophoresed on 1% agarose before being digested with S1 nuclease and Dnase I to eliminate contaminated ssDNA and ssRNA.** M1 and M2 represent the 10 and 15 kbp DNA markers, respectively.

The *S. rolfsii* strain BLH-1 produced no sclerotia when cultured on PDA for more than 6 months, whereas the other *S. rolfsii* strain, LJ-01, contained no dsRNA element that can form sclerotia, an *S. rolfsii* trait. Some abnormal phenotypic traits in plant pathogenic fungus, such as deficiency in sclerotial production, were caused by mycovirus. Therefore, we tested for mycovirus infection in this abnormal strain BLH-1. By screening for the presence of dsRNAs using the cellulose chromatography method, strain BLH-1 was found to harbor 8–10 dsRNA segments ranging in size from 1 to 15 kbp (**Figure 1**). The dsRNA extractions were confirmed to be dsRNA due to their insusceptibility to enzyme digestion with S1 nuclease and Dnase I, as well as the extraction methods through CF11 cellulose column chromatography.

### Curing of dsRNA

**FIGURE 2:**
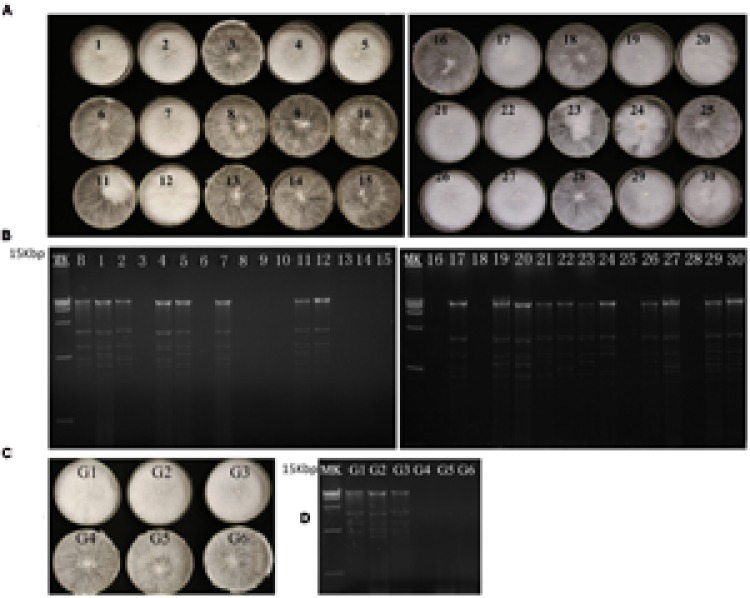
**Colony morphology and dsRNA detection results of the BLH-1 derivative isolates generated during virus curing. (A)** Colony morphology of the BLH-1 derivative isolates obtained from protoplast regeneration. **(B)** dsRNA detection of these protoplast regenerating isolates. **(C)** Colony morphology of the BLH-1 derivative isolates obtained by hyphal tip isolation up to six consecutive generations represented by G1–G6. **(D)** dsRNA extraction from the G1 to G6 derivative isolates. No dsRNA can be extracted from the fourth generation of hyphal tip isolation.

To investigate the influence of these dsRNAs in their host fungus, we conducted protoplast regeneration and hyphal tip isolation experiments to eliminate the dsRNAs from host fungus. We obtained 30 protoplast-regenerated strains. Among these, some isolates showed similar colony morphology as their paternal strain BLH-1, whereas others showed different culture traits, such as having looser hyphae growing radially on PDA. The colony morphology and the dsRNA detection results (**Figures 2A,B**) displayed a correspondence between the colony morphology of loosely growing hyphae and negative results of dsRNA presence.

The hyphal tips cut from the colony margin were subjected to culture, and sequential hyphal tips were isolated up to six generations. Colony traits in PDA became looser after hyphal tip isolation had been conducted four times (**Figures 2C,D**). The dsRNA detection also showed the elimination of dsRNA from the four-generation culture.

Surprisingly, in the virus curing experiments, all dsRNAs were consistently eliminated, either from hyphal tip culture or protoplast regeneration, as no isolate contained only partial dsRNA segments. In this study, we selected strain BLH-1-P1, a derivative dsRNA-free isolate obtained by protoplast regeneration, for further analysis.

### Presence of dsRNA Is Associated with Phenotypic Alterations

**FIGURE 3:**
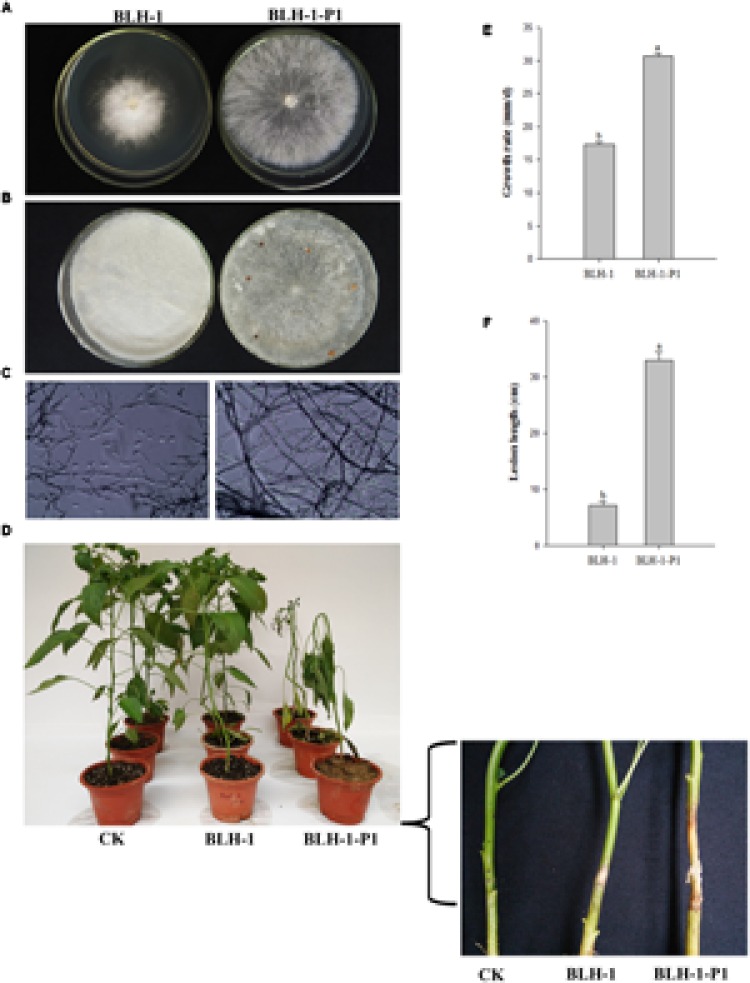
**Biological properties of *Sclerotium rolfsii* strain BLH-1 and its dsRNA-free derivative strain BLH-1-P1. (A)** Colony morphology of strains BLH-1 and BLH-1-P1 grown on PDA for 5 days at 27°C. **(B)** Sclerotia were produced in strain BLH-1 instead of BLH-1-P1 when cultured for 14 days. **(C)** Microscopy morphology examination of the 5-day-old hyphae of strains BLH-1 and BLH-1-P1. The pictures are captured at 400× magnification **(D)** Virulence assay on pepper (*Capsicum annuum*). The stems near the roots of the pepper plant seedlings were needle-pricked and inoculated by mycelial plugs. The morbidity situation was photographed after inoculation at 27°C for 5–8 days. **(E)** Average mycelial growth rates of strains BLH-1 and BLH-1-P1 on PDA at 27°C. **(F)** Average lesion length caused by strains BLH-1 and BLH-1-P1 on pepper seedling stems.

We compared the biological traits between the hypovirulent strain BLH-1 and the dsRNA-free derivative BLH-1-P1 in terms of colony morphology, growth rate, sclerotia formation and virulence (**Figure 3**).

Strain BLH-1, showing a much smaller colony size on PDA, was morphologically distinguishable from strain BLH-1-P1 (**Figure 3A**). In addition, when examined by light microscopy, the hyphal integrity of BLH-1-P1 was normal. However, the hyphae of BLH-1 were more contorted than those of BLH-1-P1 and were broken into small fragments (**Figure 3C**).

Significant differences in mycelial growth rates were detected. BLH-1 has a greater growth rate (29.83 mm/day) than BLH-1-P1 (19.12 mm/day) (**Figure 3E**).

BLH-1-P1 sclerotia initially formed after incubation for 3–4 days; brown sclerotia of 1–2 mm in diameter and with smooth surfaces were observed on each PDA plate containing almost eight grains when cultured for 8–12 days (**Figure 3B**). In contrast, no sclerotia could be found on the colonies of strain BLH-1, even when incubated for 6 months.

In the pathogenicity test, strain BLH-1-P1, within which detectable dsRNAs were lacking, caused extensive symptoms suggestive of southern blight (also called southern wilt, southern stem rot or white mold) on pepper (*Capsicum annuum*). When inoculated with BLH-1-P1, fungal threads (cobweb-like mats) expanded from the site of inoculation and rapidly covered the stem, resulting in an average lesion length of 33.00 cm. However, the dsRNA-containing strain BLH-1 merely caused small stem rot lesions confined to the inoculation site, with an average length of 7.17 cm (**Figures 3D,F**).

### Transmission of dsRNA and Hypovirulence Following Hyphal Anastomosis

We used the pairing culture technique to further confirm the effects of these dsRNA segments on the BLH-1 host and the transmissibility of the hypovirulent traits. The hyphae of the donor strain BLH-1 were fused with the recipients of BLH-1-P1, *S. rolfsii* strain LJ-01 isolated from pepper, *Botrytis cinerea* strain HM-03, and *S. sclerotiorum* strain JH-05.

**FIGURE 4:**
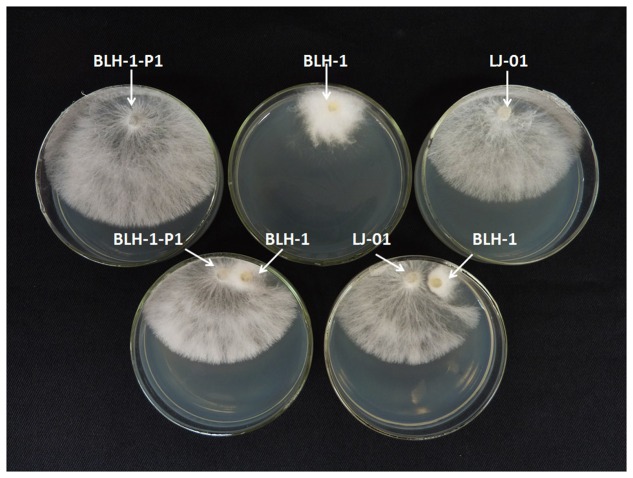
**Transmission of dsRNA elements from the hypovirulent prototype strain BLH-1 (the donor) to dsRNA-curing strain BLH-1-P1 and other wild type dsRNA- free strain LJ-01 (the recipient) on PDA using contact culture.** Mycelial plugs from the BLH-1/BLH-1-P1 and BLH-1/LJ-01 combinations were individually cultured 1 cm apart on a PDA dish. Recipient derivative isolates were obtained by picking mycelial agar plugs from the edge of each colony of the two recipients.

**FIGURE 5:**
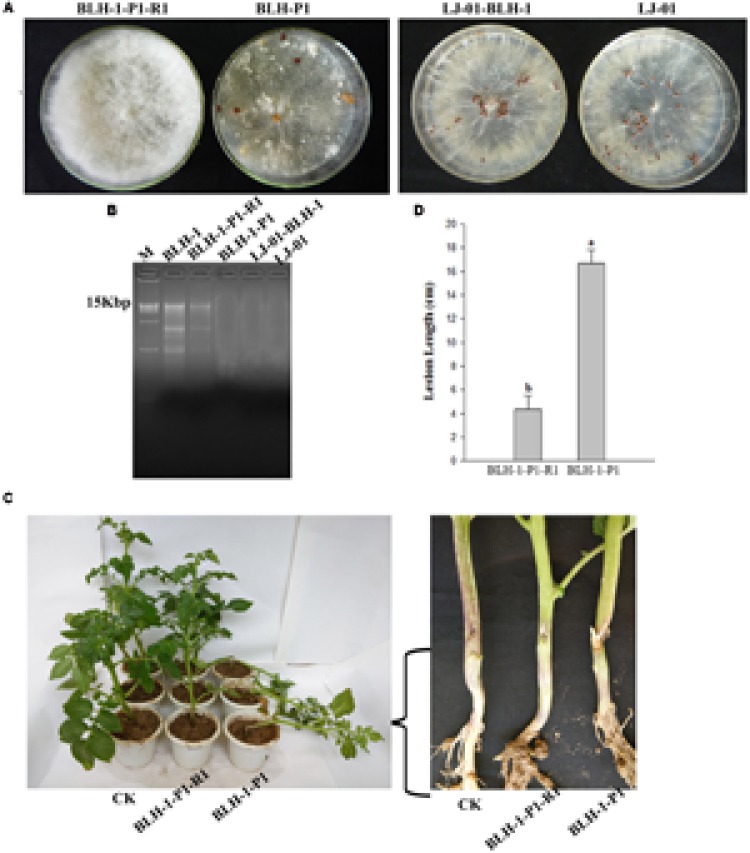
**Comparison of the biological characteristics between the recipient derivative isolates and their parental strains. (A)** Fungal colony morphology and sclerotia formation of strains BLH-1-P1, LJ-01, BLH-1-P1-R1 and BLH-01-BLH-1, the latter of which were derived from the colony margins of the recipient strains BLH-1-P1 and LJ-01, respectively. These strains were grown on PDA at 27°C for 14 days. **(B)** The results of dsRNA detection of the four strains based on 1% agarose gel electrophoresis analysis. **(C)** Virulence tests of the dsRNA-reobtaining strain BLH-1-P1-R1 and the parental dsRNA-free strain BLH-1-P1 on potato (*Solanum tuberosum*). **(D)** Lesion diameters caused by strains BLH-1-P1-R1 and BLH-1-P1 were measured after inoculation for 5–8 days.

The pairing culture experiments between *S. rolfsii* strains included five types of cultures, as shown in **Figure 4**: two double-strain contact cultures, BLH-1/BLH-1-P1 and BLH-1/LJ-01, and three single-strain cultures: BLH-1, BLH-1-P1, and LJ-01. The virulent strains BLH-1-P1 and LJ-01 grew rapidly on PDA in the single cultures, but the hypovirulent strain BLH-1 grew slowly and covered approximately one-quarter of the plate. In the contact cultures (BLH-1/BLH-1-P1 and BLH-1/LJ-01), the recipients BLH-1-P1 and LJ-01 still grew rapidly. However, a boundary formed between BLH-1 and LJ-01 in their contact culture plate, indicating a possible vegetative incompatibility reaction. Three mycelial derivative isolates of strain BLH-1-P1 and LJ-01 were obtained from the BLH-1-P1 and LJ-01 sides in each of the contact cultures. All of the dsRNA segments were detected in the derivative isolates from BLH-1-P1 but not from strain LJ-01. The dsRNAs in BLH-1 could be reintroduced to BLH-1-P1 but could not be transmitted to LJ-01 (**Figure 5B**). The representative BLH-1-P1 derivative isolate, named BLH-1-P1-R1, was similar to the hypovirulent strain BLH-1 and differed greatly from their parental strain BLH-1-P1 regarding the reduced virulence, which was tested by inoculation on potato (*S. tuberosum*) (**Figures 5C,D**), and deficient in sclerotia production (**Figure 5A**). In contrast, mycelial derivative isolates of strain LJ-01 were similar to their parental strain LJ-01, both in mycelial growth rate and colony morphology (date not shown), and can produce sclerotia (**Figure 5A**). The transmission experiments suggested that these dsRNA elements in BLH-1 could be horizontally re-introduced to BLH-1-P1 but could not be transmitted to LJ-01, and they are likely responsible for the hypovirulence phenotype of strain BLH-1.

In the pairing culture experiments between *S. rolfsii* strain BLH-1 and *B. cinerea* strain HM-03 or *S. sclerotiorum* strain JH-05, three mycelial derivative isolates of *B. cinerea* strain HM-03 and three mycelial derivative isolates of the *S. sclerotiorum* strain JH-05 were obtained from the recipient colonies of strain HM-03 and JH-05, respectively, in the contact cultures. All of the derivative isolates of HM-03 and JH-05, as well as their corresponding parental strains, showed similar colony morphology and contained no dsRNA when detected by dsRNA extraction (Supplementary Figure S1). Therefore, by hyphal contact, the dsRNAs in strain BLH cannot be horizontally transmitted to other fungal species, such as *B. cinerea* and *S. sclerotiorum*.

### Ultrastructure Examination

**FIGURE 6:**
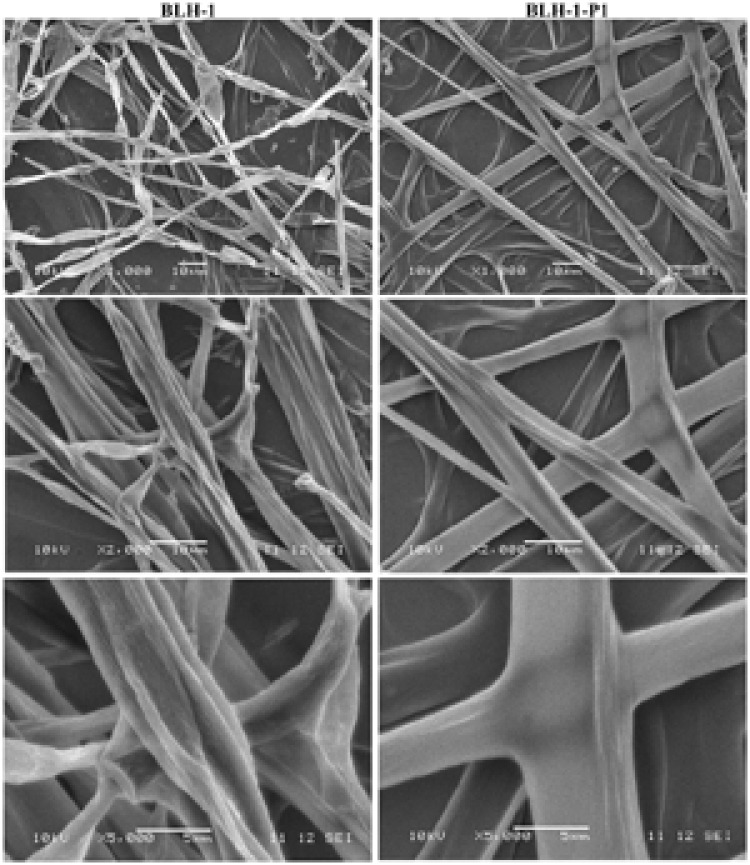
**Electron micrographs of hyphal morphology of the dsRNA-containing strain BLH-1 and dsRNA-free strain BLH-1-P1.** The (top, middle, and lower panels) are photographs captured at 1000× (scale bar: 10 μm), 2000× (scale bar: 10 μm) and 5000× (scale bar: 5 μm) magnification. The images were taken from the ultrathin sections of hyphae collected from the actively growing margins of colonies cultured for 5 days on PDA at 27°C.

Scanning electron microscopy was used to examine the ultra-micro mycelial morphology of the dsRNA-containing BLH-1 and dsRNA-free BLH-1-P1 strains. Significant differences in the mycelial morphology between the two strains were observed. The hyphae of BLH-1 become shriveled and formed fractured fragments. In contrast, the hyphae of BLH-1-P1 had a glabrate surface, which appeared to be full and integrated, displaying few small fragments. Certainly, the phenomenon of hyphae fracture was also more clearly observed by the light microscope using microscopic sections from 5-day-cultured hyphae (**Figure 6**).

**FIGURE 7:**
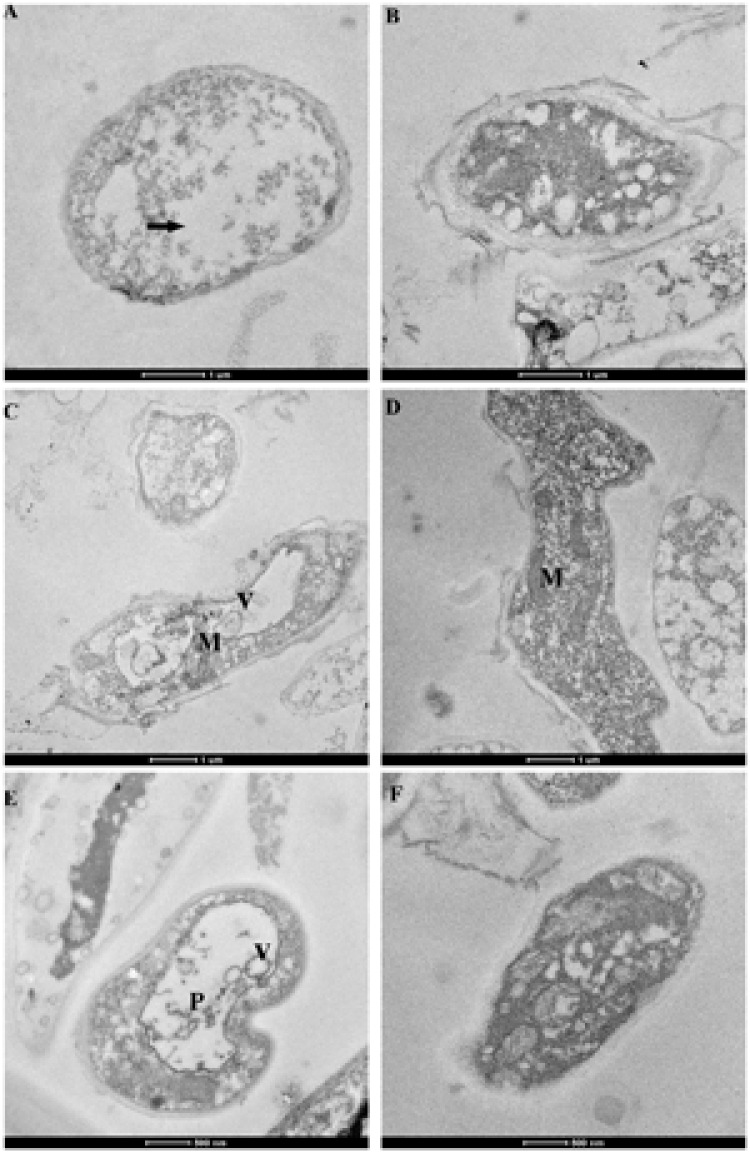
**Cytoplasmic characteristics of the dsRNA-containing hypovirulent strain BLH-1 (A,C,E)** and the dsRNA-free virulent strain BLH-1-P1 **(B,D,F)** examined by TEM. **(A)** Hyphal cells of BLH-1 were degenerated, showing electron-sparse and unevenly distributed cytoplasm, indicated by arrow. **(C)** BLH-1 had significantly fewer mitochondria (indicated by M) with swollen shape, whereas BLH-1-P1 **(D)** contained numerous oval or oblong mitochondria. **(E)** Virus-like particles with sizes under 100 nm (indicated by P), and abundant vacuole-like membranous structures or small membranous vesicles (indicated by V) were observed in the hyphal cells of BLH-1. **(F)** Strain BLH-1-P1 shared dense and evenly distributed cytoplasm as that shown in **(B)**. Note: scale bars for pictures **(A–D)** were 1 μm and for **(E,F)** were 500 nm.

Ultrathin hyphal sections for each strain of BLH-1 and BLH-1-P1 were observed under TEM (**Figure 7**). Comparison of the ultrastructure of hyphal cells showed that the cytoplasm and mitochondria differed greatly between the two strains. The cytoplasm of BLH-1 was degenerated and formed vacuole-like membranous structures and small membranous vesicles, similar to the hypha of some mycovirus-infected fungi. Moreover, virus-like particles with a size under 100 nm were observed in the hyphal cells of BLH-1 (**Figure 7E**). In comparison, the virulent isolate BLH-1-P1 exhibited a relatively electronically dense and evenly distributed cytoplasm, with fewer membranous vacuoles or vesicles formed in hyphal cells (**Figures 7B,D,F**). Ultrastructure of mitochondria showed that BLH-1-P1 contained numerous mitochondria with normal oval or oblong shape (**Figure 7D**). The mitochondria in BLH-1 were significantly fewer and became swollen, exhibiting degradation syndrome (**Figure 7C**).

### Enzyme Activity Assays

**FIGURE 8:**
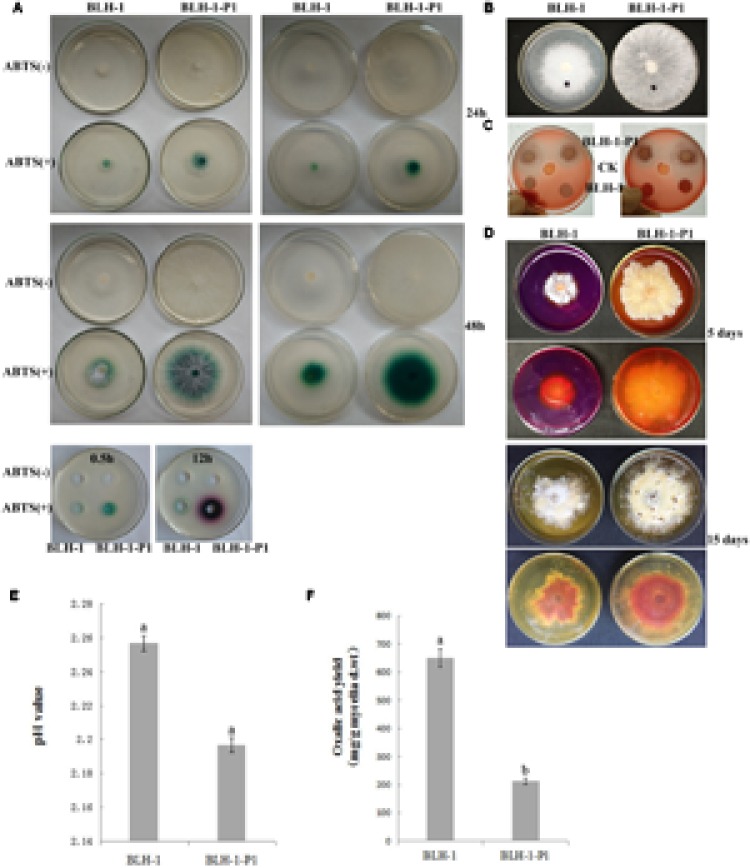
**Assays for laccase activity, cellulase activity, acid-producing ability, ambient pH, yield of oxalic acid and the colony surface hydrophobicity. (A)** Laccase activity was tested after placing mycelial agar plugs on PDA containing ABTS for 24 and 48 h. It was also assayed using mycelial culture filtrate dropped upon holes on the same PDA agar. The images were collected after the culture filtrates were dropped for 0.5 and 12 h. **(B)** Colony surface hydrophobicity test using bromophenol blue. **(C)** Cellulase activity assays of the two strains by examination of the formation of bright yellow color around the fungal colonies. **(D)** Acid-producing ability was evaluated by culturing the mycelial agar plugs on PDA containing 0.01% bromophenol blue for 5 and 15 days, respectively; acid production was indicated by appearance of yellow in the culture medium. **(E)** pH values of cultures of strains BLH-1 and BLH-1-P1 that inoculated in PD for 5 days. **(F)** The amount of oxalic acid produced by the strains of BLH-1 and BLH-1-P1 were assessed by high-performance liquid chromatography.

Some reports suggested that laccase activity might be involved in fungal virulence (Ahn and Lee, 2001). We compared the laccase activities between strains BLH-1 and BLH-1-P1. The two strains growing on PDA plates containing ABTS produced a green color reaction, indicating the presence of laccase activity in each culture. However, the color reaction was more clearly observed in strain BLH-1-P1 than in the virus-infected strain BLH-1. When assayed using mycelial culture filtrate dropped upon holes on the ABTS containing PDA plates, the virus-cured strain BLH-1-P1 also showed higher laccase activity (**Figure 8A**). As the hydrophobicity of a fungal colony may reflect the cell wall integrity of fungal hyphae (Yu J. et al., 2015), we used a 15-μl droplet of water containing bromophenol blue to measure the hydrophobicity of the hyphal surfaces of the two strains. Unexpectedly, spherical droplets formed on both strains, and there was no significant difference in hydrophobicity between the two strains (**Figure 8B**). To examine cellulase activities, the transparent circles around the fungal colonies, which indicate the capacity to degrade carboxymethyl cellulose, were obvious in BLH-1-P1 but not apparent in BLH-1 colonies (**Figure 8C**).

The PDA containing 0.01% (wt/vol) bromophenol blue became yellow during hyphal extension of the two strains after they had been cultured for 5 days. However, the degree of yellow in strain BLH-1-P1 was significant larger and obvious than that in strain BLH-1 (**Figure 8D**). Because the yellow would form if the pH decreased in the bromophenol blue-containing PDA, we can infer that both strains produced acid, with the virus-cured strain BLH-1-P1 producing more at this time point. However, the PDA of the two strains become yellow when cultured for a longer time (**Figure 8D**). The result indicated that both the two *S. rolfsii* strains of BLH-1 and BLH-1-P1 have the ability to produce acid, and the difference in acid-producing ability between the two strains in different culture time might be ascribed to difference in mycelial growth rate. The pH value of BLH-1 was slightly larger than that of BLH-1-P1(2.26 vs. 2.20) (**Figure 8E**), the oxalic acid yield of strain BLH-1 (649.76 mg/g of dry mycelia) quantitative assayed from the 5-day-old cultures grown in PD was significantly higher than that of strain BLH-1-P1 (212.74 mg/g) (**Figure 8F**).

### Partial Sequence Analysis of the dsRNAs Associated with BLH-1

A cDNA library was synthesized using dsRNAs from the hypovirulent strain BLH-1. After the first round of sequencing, sequence-specific primers were designed and used to fill the gaps between the cDNA clones. Consequently, three assembled cDNA contigs were obtained, named contig 1, contig 2, and contig 3. The nucleotide sequences of the contigs have been deposited in GenBank under the accession numbers KU885931, KU885932, and KU885933.

**FIGURE 9:**
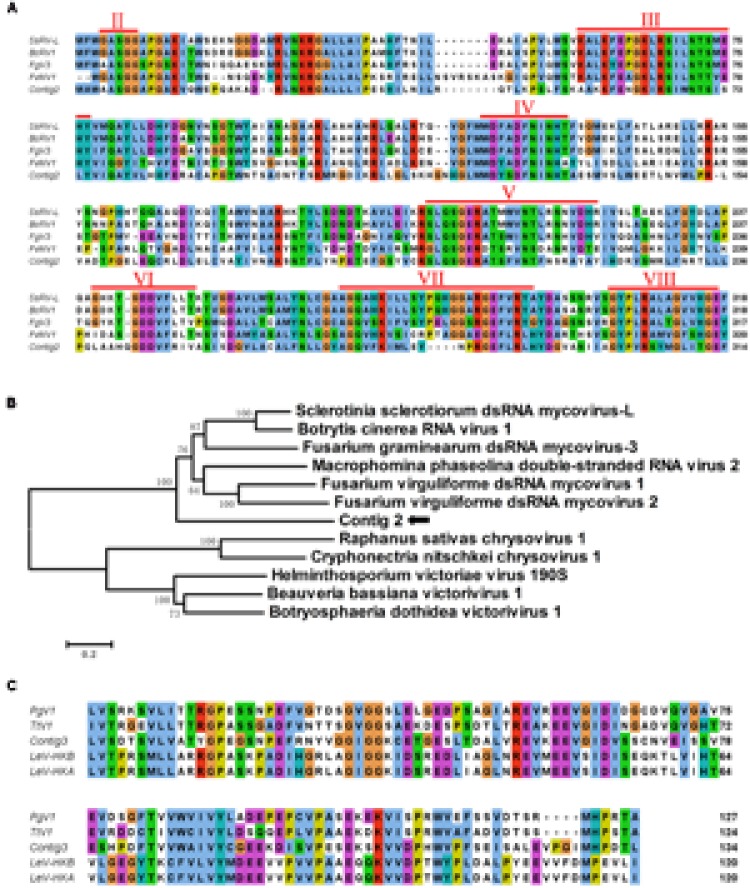
**Multiple alignment of contig 2 and contig 3 translated amino acid sequences and phylogenetic analysis of the contig 2 encoded protein. (A)** Multiple alignment of the viral RNA-dependent RNA polymerases (RdRp) encoded by contig 2 and other similar dsRNA mycoviruses. Conserved sequence motifs (denoted by numbers II to VIII) characteristic of the RdRps of dsRNA mycoviruses are indicated. The alignment was prepared by the program CLUSTAL_X and viewed by Jalview. **(B)** A phylogenetic tree was constructed based on the alignment of the contig 2-encoded RdRp and selected viruses. The phylogenetic analysis was conducted with the neighbor-joining method using MEGA 6, with bootstrap of 1000 replicates. **(C)** The NUDIX domain was shown by multiple alignments of aa sequences of contig 3 and its related mycoviruses. The viral names and accession numbers for the above analysis are as follows: SsRV-L, Sclerotinia sclerotiorum dsRNA mycovirus-L (CEZ26307.1); BcRV1, Botrytis cinerea RNA virus 1(YP_009115498.1); FgV3, Fusarium graminearum dsRNA mycovirus-3 (YP_003288789.1); FvMV1, Fusarium virguliforme dsRNA mycovirus 1 (AEZ54148.1); FvMV2, Fusarium virguliforme dsRNA mycovirus 2 (AEZ54146.1); RsSCV1, Raphanus sativas chrysovirus 1 (AFE83590.1); CnCV1, Cryphonectria nitschkei chrysovirus 1 (ACT79256.1); HvV 190S, Helminthosporium victoriae virus 190S (NP_619670.2); BbV1, Beauveria bassiana victorivirus 1(CCC42235.1); BdV1, Botryosphaeria dothidea victorivirus 1 (YP_009072433.1); PgV1, Phlebiopsis gigantea mycovirus dsRNA 1 (YP_003541122.1); TtV1, Thelephora terrestris virus 1(YP_009209481.1); LeV-HKB, Lentinula edodes mycovirus HKB (BAG71789.2); LeV-HKA, Lentinula edodes mycovirus HKA (BAM34027.1).

**FIGURE 10:**
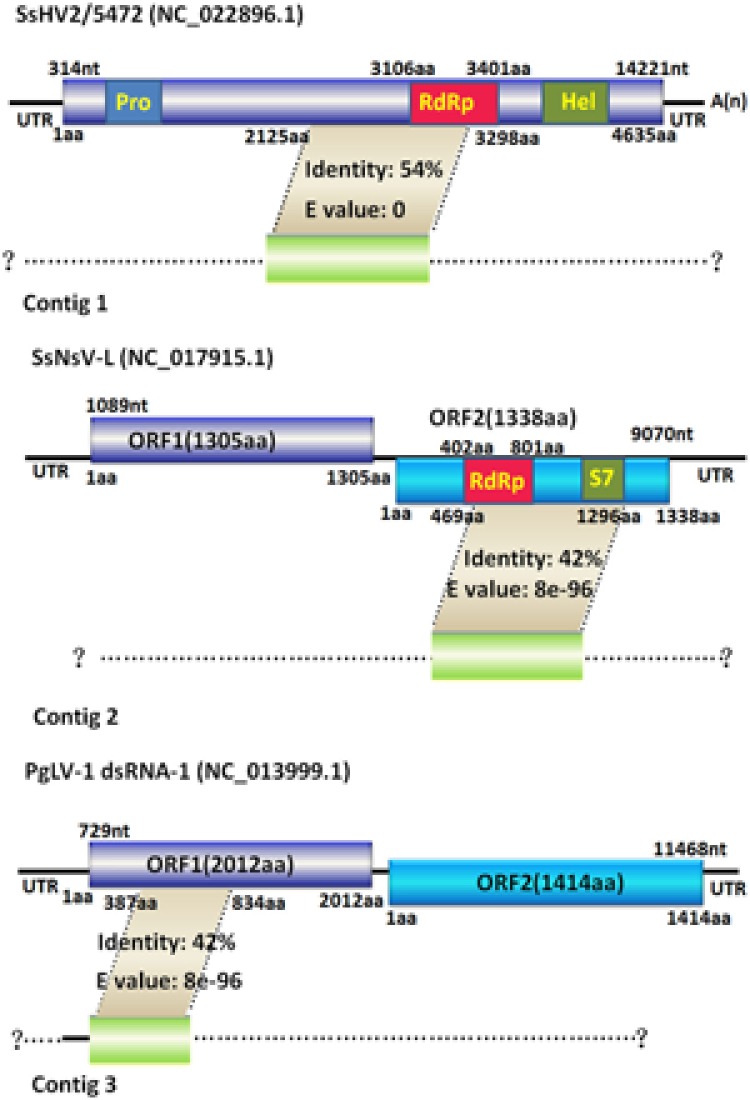
**Genomic organization of the three contigs and their comparison with each similar reported mycovirus.** Contigs 1, 2, and 3 are schematically represented in the (top, middle, and lower panel), respectively. Contigs 1, 2, and 3 have maximum amino acid sequence similarities to SsHV2/5472, SsNsV-L and PgLV-1 dsRNA1, respectively. The dashed lines indicate the possible undetermined sequences of the three contigs’ corresponding viruses. The colored boxes and lines represent ORFs and non-coding sequences (UTR), respectively. The smaller boxes in the SsHV2/5472 ORF and the SsNsV-L ORF2 showed conserved domains, such as Pro, RdRp, Hel and S7. The nt and aa numbers above the boxes indicate the map positions corresponding to their genomic structure. The gray areas between the three contigs and their respective most closely related mycoviruses denote the homologous regions with similarities to the translated amino acid sequences of the three contigs.

Contig 1 (3558 bp) was predicted to encode a protein with similarity to members of the family *Hypoviridae*. Significant hits included Sclerotinia sclerotiorum hypovirus 2 (SsHV2), Macrophomina phaseolina hypovirus 1 and Cryphonectria hypovirus 1; SsHV2 had the maximum amino acid similarity of 54% at query coverage of 99%. We suggested that contig 1 likely represented the partial genome sequences of a novel hypovirus related to SsHV2. Contig 2 (2781 bp) had sequence identities ranging from 32 to 33%, with query coverage of more than 90%, to the putative RNA-dependent RNA polymerase of Sclerotinia sclerotiorum dsRNA mycovirus-L (SsNsV-L) (Liu et al., 2012), Fusarium graminearum dsRNA mycovirus-3 (FgV3) (Yu et al., 2009) and Botrytis cinerea RNA virus 1(BcRV1) (Yu L. et al., 2015). Multiple protein sequence alignments and comparisons demonstrated that contig 2 encoded a protein with an RdRP_4 superfamily domain (cl19931) that contained conserved motifs characteristic of the RdRps of dsRNA mycoviruses (**Figure 9A**). Thus, we proposed that contig 2 belongs to a novel mycovirus. Phylogenetic analysis showed that contig 2 clustered with SsNsV-L, FgV3, BcRV1 and other unassigned dsRNA mycoviruses, forming a clade distinct from the *Totiviridae* and *Chrysoviridae* clade (**Figure 9B**). Contig 3 (2449 bp) was identified with similarity to the hypothetical proteins of unclassified dsRNA mycoviruses, including Phlebiopsis gigantea mycovirus dsRNA 1 (PgV1) (Kozlakidis et al., 2009), Thelephora terrestris virus 1, Lentinula edodes mycovirus HKB (Lev-HKB) (Magae, 2012) and Lentinula edodes mycovirus HKA (LeV-HKA), displaying identities of 27–43% with query coverage of greater than 94%. LeV-HKB is a dsRNA mycovirus, with an 11-kbp monopartite genome identified from *Lentinula edodes*, belonging to basidiomycetes. The genome of this virus contains two ORFs, with the 5′ proximal ORF (ORF1) encoding a hypothetical protein and the 3′ proximal ORF (ORF2) expressing a putative RdRp. Based on the similarities between the predicted protein of contig 3 and the ORF1-encoded hypothetical proteins of the Lev-HKB-like mycoviruses, together with the presence of the NUDIX domain (**Figure 9C**) (Parrish et al., 2007), which was conserved in Lev-HKB and the contig 3-encoded proteins, we suggest that contig 3 represents the 5′ half of a novel mycovirus closely related to the Lev-HKB-like mycoviruses. A comparison of the genomic organization between the three contigs and their respective most closely related viruses is shown in **Figure 10**.

## Discussion

*Sclerotium rolfsii* Sacc. is a versatile soil-borne plant pathogen that causes Southern blight diseases on more than 600 plant species. This pathogen produces sclerotia, which can survive for a long time in the soil, as the primary inoculum source for disease development, making this disease a severe problem (Punja, 1985). Thus, either reducing the sclerotia population in the soil or preventing the infection of host plants is critical for Southern blight control. In this study, we provided the first evidence of hypovirulence caused by dsRNA elements in *S. rolfsii.* The presence of hypovirulence was demonstrated by hyphal tip isolation and transmission tests. The dsRNA-containing strain BHL-1 grew slowly and had debilitated virulence compared to dsRNA-free isogenic *S. rolfsii* isolates, which were obtained by hyphal tipping or protoplast regeneration. The dsRNA-free isolate exhibited a faster mycelial growth rate and became virulent on potato (*S. tuberosum*) and pepper (*C. annuum*). Moreover, the dsRNA acquisition of the dsRNA-free *S. rolfsii* isolate through hyphal anastomosis transmission restored the wild-type dsRNA-containing hypovirulent phenotype. These results provided clear evidence that the dsRNAs in the BHL-1 strain confer hypovirulence to *S. rolfsii* displayed mainly by reduced mycelial growth on PDA and pathogenicity on two tested plants, along with deficiency in sclerotia production.

In our study, based on virus curing and transmission tests, we confirmed that dsRNA elements have the ability to reduce the pathogenicity of *S. rolfsii.* Concurrently, several enzyme activities such as laccase and cellulose and oxalic acid production, which are all closely related with pathogenicity, were reduced in dsRNA-containing strain BLH-1 compared with virus-free strains. As reported previously, the production of laccase and cell wall degrading enzymes associated with fungal pathogenicity were reduced by mycovirus infection, such as the laccase activity decrease in *C. parasitica* caused by CHV1 infection. Castro et al. (2003) reported that the pathogenicity, conidiation and laccase production in the dsRNA-infected hypovirulent *Botrytis cinerea* strain were reduced to 50% compared to that in the virus-curing *B. cinerea* strain. During *S. rolfsii* infection, hyphae penetrate host tissue by formation of appressoria and with the aid of phytotoxins such as oxalic acid and cell wall degrading enzymes (pectinolytic and cellulolytic enzymes) (Bateman and Beer, 1965), which were the main pathogenic factor of *S. rolfsii.* Therefore, we can attribute the hypovirulence of *S. rolfsii* to the reduced production of pathogenic factors of *S. rolfsii* caused by the infected dsRNA elements. Nevertheless, the exact regulatory mechanism of the pathogenicity-related gene of *S. rolfsii* remains to be elucidated.

Compared to dsRNA-free BLH-1-P1, which showed an integrated hyphal structure, the hyphae of dsRNA-containing BLH-1 became shriveled, constricted and fractured into small fragments. We speculated that the damage to the hyphal structure consequently gave rise to the reduced infection ability of this pathogenic fungus. The ultrastructure of cellular cytoplasm in the hypovirulent strain was highly degenerated, as has been reported for other mycovirus-infected fungus, with the appearance of abundant vacuole-like membranous structures and disappearance of cellular organelles. In addition, mitochondria were rare and malformed. Moreover, some membrane-bound vesicles of less than 100 nm in diameter can also be found by TEM, which might represent the area formed by host membranes. Coincidently, the CHV1-infected fungal cells also contain pleomorphic lipid vesicles with diameters of 50–80 nm (Fahima et al., 1993; Suzuki and Nuss, 2002), and the virus has been confirmed to be packaged in membranous vesicles and the trans-Golgi network (TGN) for replication (Jacob-Wilk et al., 2006). Similar membrane structures were also examined in the *S. sclerotiorum* cytoplasm transfected with SsHV2L, a recombinant of SsHV2 (Marzano et al., 2015). These membranous vesicles in virus-infected fungal cells may have the ability to protect the viral nucleic acid from degradation by host nucleases (Mackenzie, 2005). Therefore, the membrane-bound vesicles in BLH-1 may possess the same function for virus replication and protection as in other hypovirus-infected fungi. Mitochondria are extremely important organelles for the energy generation used for cellular activities. Previous reports have shown that the fungal mitochondria were targeted by their infected mitoviruses and become ultrastructurally malformed, thus leading to the debilitation of plant pathogenic fungus (Park et al., 2006; Wu et al., 2010). Rogers et al. (1987) reported that the hypovirulence of *Ophiostoma novo-ulmi* resulted from a deficiency in cytochrome aa3 caused by mitoviruses that targeted the mitochondria of this plant pathogenic fungus. Although at present we cannot determine if there is any mitovirus infection in BLH-1, the mitochondrial malformations in *S. rolfsii* may contribute to the hypovirulence of this fungus.

Oxalic acid has been considered as a key pathogenicity factor utilized by some plant pathogenic fungi, such as the *S. sclerotiorum*, to rapidly kill host cells and tissues as well as to suppresses host oxygen burst, triggers ROS mediated programmed cell death (Dickman and Mitra, 1992; Criscitiello et al., 2013; Dickman and Fluhr, 2013). In *Botrytis cinerea*, an oxalate-deficient mutant strain also has reported to be non-pathogenic to plants (Kunz et al., 2006). However, in our study, the amount of oxalic acid produced by the hypovirulent dsRNA-containing strain BLH-1 was not inhibited, and was qualitative detected to be larger than that of the dsRNA-free BLH-1-P1. Therefore, we can hypothesis that the oxalic acid production might not be the decisive pathogenicity factor of *S. rolfsii.* Actually, this complexity is not unusually, i.e., a T-DNA inserted secretory protein Ss-Caf1 mutant and a targeting silenced SSITL mutant of *S. sclerotiorum* were all reported to be less virulent, but produced significant amounts of oxalic acid (Zhu et al., 2013; Xiao et al., 2014); the oxalic acid production of a hypovirulent *B. cinerea* strain CanBc-1 that mediated by mycovirus was also reported to be higher than that for other virulent strains (Zhang et al., 2010). Thus, it seems plausible liking those have been proven in *S. sclerotiorum* that they have other factors involved in pathogenesis of *S. rolfsii.* However, to uncover the pathogenesis-related gene and the contributions of oxalic acid to pathogenicity in *S. rolfsii*, more studies remain needed.

In this study, culturing the dsRNA-containing hypovirulent *S. rolfsii* strain BLH-1 and the dsRNA-free strain BLH-1-P1 for 15 days on PDA containing 0.01% (wt/vol) bromophenol blue indicated that all the two strains produced acid and have the ability to reduce the ambient pH. This could be proven by detection of the pH of the two fungal cultures grown in PD broth for 5 days, within which BLH-1 and BLH-1-P1 strains showed the similar lower pH lever. In addition, the amount of oxalic acid (mg/g dry mycella) produced by the hypovirulent dsRNA-containing strain BLH-1 was qualitative detected to be three times larger than that of the dsRNA-free BLH-1-P1 when the two strains were cultured for 5 days in PD. Nevertheless, when the two strains were cultured for only 5 days on bromophenol blue contained PDA, the acid production of BLH-1 was lower than that of BLH-1-P1. This discrepancy was also reflected from the results of pH detection in PD broth clutures exhibiting that the pH of BLH-1 was slightly higher than that of BLH-1-P1 when cultured for 2 and 3 days, but slightly lower than that of BLH-1-P1 once cultured for exceeding time of more than 5 days (date not shown). This might be explained that the amount of oxalic acid production was correlated with the amount of mycelium mass. Although the amount of oxalic acid produced (mg/g dry mycella) by the hypovirulent BLH-1 was larger than that of BLH-1-P1, whereas the slower growth rate of BLH-1 at first few culture days resulted in a relatively less production of oxalic acid in total. With the extension of incubation time and the increasing of mycelia amount, the yield of oxalic acid in the PD cultures of BLH-1 will increase and exhibit a lower PH.

BLH-1 lost the capacity to produce sclerotia. The formation and development of sclerotia are associated with a series of signaling genes, such as reports in *S. sclerotiorum* that the microbial opsin homolog gene sop1 was involved in sclerotial development and virulence of this fungus (Lyu et al., 2015). Previous studies have shown that sclerotial development in some fungi was associated with and affected by a series of physical, chemical or nutritional factors, such as low temperatures, oxidative stress and low PH (Chet and Henis, 1975; Choi et al., 2002; Xing et al., 2013). In addition, the cAMP and the mitogen-activated protein kinase (MAPK) signal pathways are involved in sclerotial development of *S. sclerotiorum* (Rollins and Dickman, 1998; Chen et al., 2004), and this process also requires calcineurin in an oxalic-acid-independent manner (Harel et al., 2006). In *S. rolfsii*, sclerotial differentiation was proven to be closely related to a high degree of lipid peroxidation evoked by oxidative stress, and the sclerotial development could be inhibited by antioxidants such as β-carotene (Georgiou, 1997; Ellil, 1999; Georgiou et al., 2001). In *S. sclerotiorum*, oxalate production was previously assumed to be involved in sclerotial development (Dickman, 2007). Later, Xu et al. (2015) confirmed that it was the acidified conditions of low pH, which might be caused by oxalate production, not oxalic acid itself, that necessary for virulence and sclerotial development of *S. sclerotiorum.* Since the BLH-1 has the ability to produce an amount of oxalic acid and reduce the ambient pH, thus we can propose that other factors independent of oxalic acid and pH that were influenced by mycovirus infection in BLH-1 might involve in sclerotial production. Recently, oxalic acid was demonstrated to inhibit sclerotial formation of *P. umbellatus* (Xing et al., 2015). However, whether the sclerotia production deficiency of *S. rolfsii* was caused by the excessive production of oxalic acid that might inhibit sclerotial formation in this fungus or through blocking of other unknown pathways remains to be clarified. Besides, the molecular mechanism of sclerotial development of *S. rolfsii* is unclear. Therefore, it will be of interest and feasible to use the hypovirulent *S. rolfsii* strain as a model to investigate the sclerotial formation mechanism and its effect in pathogenesis. Moreover, because they are the primary inoculum source for disease epidemiology, this deficiency might be harmful for pathogen spread but favor the control of this pathogen. Interactions between some hypovirulence-associated viruses and their hosts have contributed to the establishment of some host-mycovirus systems, by which we can better understand the molecular basis of fungal biology, especially for fungal pathogenesis (Xie and Jiang, 2014; Wang et al., 2015). These host-mycovirus systems, including Cryphonectria parasitica-hypovirus (Nuss, 1996, 2011; Dawe and Nuss, 2001, 2013; Hillman and Suzuki, 2004; Milgroom and Cortesi, 2004; Nuss, 2005; Pearson et al., 2009), Helmintosporium victoriae-HvV190S (Li H. et al., 2011; Dunn et al., 2013; Ghabrial et al., 2013), Sclerotinia sclerotiorum-mycovirus (Li et al., 2008), Rosellinia necatrix-mycovirus (Salaipeth et al., 2013; Yaegashi et al., 2013), and Fusarium graminearum-mycovirus (Kwon et al., 2009; Cho et al., 2012), have been established and thoroughly elucidated, providing plenty of references to study fungal pathogenicity (Xie and Jiang, 2014). The isolation of the hypovirulent *S. rolfsii* strain displaying a series of abnormal phenotypic traits would be useful for further studying the biology of this plant pathogen, including the pathogenesis and mechanism of sclerotial formation, which we can target to reduce yield loss caused by this plant pathogenic fungus. To identify *S. rolfsii* host factors involved in abnormal host phenotypic traits or response to mycovirus infection, the interaction system at both the proteomic and transcriptional levels should be established.

Many mycoviruses in the families *Hypoviridae*, *Megabirnaviridae*, *Narnaviridae*, *Partitiviridae*, and *Reoviridae*, the unassigned ssRNA, and ssDNA mycoviruses that belong to the newly established family *Genomoviridae* have been reported to cause attenuated symptoms on the host fungi (Nuss, 2005; Chiba et al., 2009; Pearson et al., 2009; Yu et al., 2010; Liu et al., 2014; Krupovic et al., 2016). Moreover, there is a large number and diversity of mycoviruses showing divergent molecular characteristics in nature. Thus, it is difficult to estimate the viral species by using electrophoretic profiles of dsRNA elements. In this study, we obtained three assembled sequence contigs, which might belong to the genome sequences of three different mycoviruses, through cDNA library synthesis using the total dsRNAs extracted from BLH-1 as templates. The results indicated that at least three mycoviruses co-infected the hypovirulent strain BLH-1. Although at present we could not definitely determine the numbers and species of mycoviruses infecting BLH-1, as well as which virus confers hypovirulence to the host fungus, some clues are provided in view of the possible effects imposed on the fungal host by these mycoviruses that are closely related to our obtained sequence contigs. Contig 1 was most closely related to a *S. sclerotiorum*-infecting hypovirus, SsHV2. Evidence was presented in three recent reports that SsHV2 and its recombinant strains are responsible for hypovirulence of *S. sclerotiorum* (Hu et al., 2014; Khalifa and Pearson, 2014; Marzano et al., 2015). In addition, Marzano et al. (2015) proposed that there was a symptom determinant associated with sclerotia production in the SsHV2 genome, according to genome structure comparison between the deleted SsHV2L, SsHV2/5472, and recombinant SsHV2L. We hypothesize that BLH-1 was infected by a putative novel hypovirus related to SsHV2. There was no example of contig 2-related Lev-HKB-like mycoviruses causing hypovirulence, and the phenotypic effects, especially for pathogenicity caused by contig 3-related SsNsV-L-like mycoviruses, were also diverse. Therefore, we hypothesized that the hypovirulence of BLH-1 was mainly ascribed to the infection of contig 1-associated hypovirus. Eventually, the attenuated phenotype of BLH-1 was similar to the SsHV2/SX247-infected *S. sclerotiorum* strain, which displayed a complete loss of sclerotia production and reduced pathogenicity (Hu et al., 2014). Nevertheless, whether the BLH-1 hypovirulence was caused directly by the contig 1-associated hypovirus or by combinations of multiple mycoviruses or satellite RNA is unclear and should be elucidated further, as other unknown mycoviruses that have not yet been cloned in cDNA library synthesis may exist in this fungus.

In our virus elimination and transmission experiments, all of the dsRNAs that belong to different mycovirus species were co-instantaneously eliminated or transmitted, demonstrating that the dsRNAs in BLH-1 harbor similar stability and equal transmission rates, resembling other mixed-infected viruses in *Epichloë festucae*, showing the consistent transmission rate to conidia (Romo et al., 2007). In addition, co-infection of multiple mycoviruses and satellite RNA or defective RNA, if any, in one fungal strain may give rise to potential interactions such as additive or synergistic effects between the mycoviruses. For example, a mutual interplay between mix-infected mycovirus has been reported for capsidless (+) ssRNA virus, yado-kari virus 1 (YkV1), which can hijack the capsid protein of a dsRNA virus, yado-nushi virus 1 (YnV1), for trans-encapsidation of not only its RNA but also the RdRp. The virus can replicate like a dsRNA virus and enhance YnV1 accumulation as well (Zhang et al., 2016). In addition, some mycoviruses rely on other co-infected mycoviruses or satellite RNA to cause host hypovirulence, such as Rosellinia necatrix megabirnavirus 2 (RnMBV2), which confers hypovirulence in *Rosellinia necatrix* only when another partitivirus was co-infected in the host fungus (Sasaki et al., 2016). Other mycoviruses including Sclerotinia sclerotiorum botybirnavirus 1 (SsBRV1) and Sclerotinia sclerotiorum hypovirus 1 (SsHV1/SZ-150) harbored satellite RNA as their requirements to cause hypovirulence (Xie et al., 2011; Liu et al., 2015). To fully determine the species and genomes of all of the mycoviruses infecting BLH-1, which could facilitate the construction of a full-length infectious cDNA clone of each viral genome that is necessary for the establishment of the cause-and-effect relationship between individual mycoviruses and their host, as well as clarify the possible interactions between these co-infected viruses, further sequencing study is needed and underway.

In summary, we confirmed in this study that BLH-1 was a hypovirulent strain and that the hypovirulence was positively correlated with its associated dsRNA elements. To our knowledge, this is the first report of hypovirulence caused by mycovirus in *S. rolfsii*, which may not only provide novel insights for the study of molecular mechanisms underlying the pathogenesis of this plant pathogenic fungus but also enrich virocontrol resources for southern blight disease.

## Author Contributions

JZ and QZ conceived and designed the experiments; DC performed the experiments; HZ and BG analyzed the data; JZ wrote the paper.

## Conflict of Interest Statement

The authors declare that the research was conducted in the absence of any commercial or financial relationships that could be construed as a potential conflict of interest.

## Footnotes

1 http://www.ncbi.nlm.nih.gov/genomes
