# Supplementary material for: Hypovirulence of Sclerotium rolfsii Caused by Associated RNA Mycovirus
Source: Front Microbiol. 2016 Nov 10;7:1798. doi: 10.3389/fmicb.2016.01798 (PMC5103162; doi:10.3389/fmicb.2016.01798)
Supplement: Supplementary file 1 [file Data_Sheet_1.DOCX]

Fig.S1


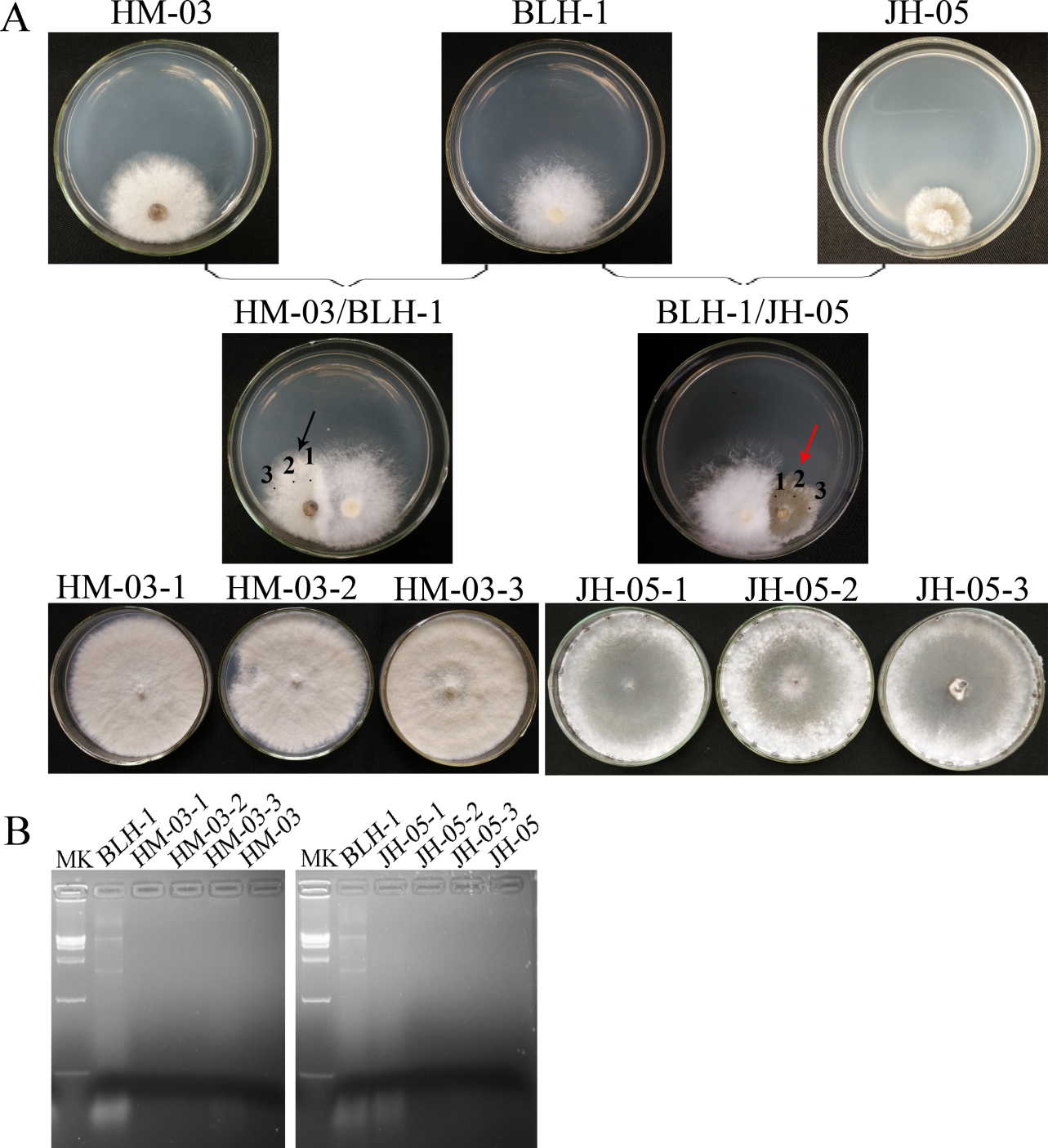


**FIGURE S1 Pairing culture experiments for transmission of dsRNAs from hypovirulent strain BLH-1 to *B. cinerea* strain HM-03 or *S. sclerotiorum* strain JH-05. (A)** The top panels showed the single cultures of BLH-1, HM-03 and JH-05, and the middle panels showed the pairing culture of BLH-1/HM-03 and BLH-1/JH-05 on PDA. The lower panels indicated the mycelial derivative isolates of HM-03 and JH-05 obtained by picking mycelial agar plugs from the recipient colonies of HM-03 and JH-05 in their respective pairing cultures of BLH-1/HM-03 and BLH-1/JH-05. Note the marked dark spot in the HM-03 and JH-05 of the pairing cultures denoted the locations where mycelial agar plug were removed. **(B)** DsRNA extraction from the derivative isolates of HM-03 and JH-05 for detection of dsRNA transmission. Except for donor strain BLH-1 no dsRNA was detected in these derivative isolates.

Nucleotide sequences of the assembled contigs

> Contig 1

CGAGCAGCGGCTGTCGCGGGGGACTTTGGTCTTGAGGGCATTCGAGTGCCCAGACTAACCAGTCCTTTCGACAAGCCCCCGACTCCGCGGAAGGACACGCTCCTCATCACGGTTCTCGGCTCATACGGAGACTTAGTCCCCATGGAGTACCTGGCGCGAGTCATCATCGCCTCTGGGGTTCCTGCGGTGTTATGGGTGACCCAAACACTAGAAGCAAGTGACTTCGACCAGCTGAAGGCAGGCAACGTGATCCGGGCTCTGCCCGGCTTCCTGGCGGCTATCGGCGCCCACAACCTCGGATATAAGGCAGTTCTAGCCCCTAACTTCCGGATGTCGCTCAACACCATCAAGTATACTTTGGCTAACTGGGAAACGACTGCCCGGCCTTCGTTGGTGGCGAATCCCCAAGCTTGGCTGGCAGCCTTCTTGATCCAATACGTCGACAAAGGGCTGGTCATCGGCAACACTCCAGGGTGCAACACCCCACGCTCAGCAAACGGACGGACCATGTTGCTTTCTCAGCAACCAGCCCAGACTCGTCGCCCGTGCGCCTGGGTCGAATGTTCTGACGGTGTCCAAGCGGTTCCGATGTCCATCAGGAACTCGATGCCTGGCATCACACGGCCTTATGATCCTTCATGTTTCTGCGAGTACGAGGTGATTTATTGCTCCGGATCCCAGGGTGTGGTCGACACGATCCTCGCTCATGGAGCGAAAGCAATGCTGTTCGACGCGTGGTTCGACCAGAAACGGACGATAGCTCTGGTCCCGGAGGTGTTCACTGAGCCCACCTGGCAGGCACTTGGGGAGGCACTCTATCATCACGGATTTGAGAGTACGCTTCCCAAGCCCAGCTGGTCCAGACGTTACACTCAGAGGATCCCAAATCTTCGACAGGGTCTGCGTTCGGTAATTACGCTCGTGACCACCAGCCAGTTGCTCCTCGCGTTGGCTCCATGGTTCGGCACTTTCCCGAGTCTTGCTCTTGAGTGGCCAGGACGACTTGCATTGCTCCATCCGGTCATGAGGACATTGATGAACGTTCCTCTGTTGTACTCCTGGTGTGGAACAAAGAGCGTTGCTTACCTCATCATCTTCTGGTATCTCTTTGAGCAGATCCCTCTGGTCGCTCTTGGGATGCAGGAGGGTGTGAAGCTCCGCCTCGAACTCAACCCAGGTTCATGGCTCTTCGTCCGACACGCGTCCTTGGTACACACCAAGAAGAAACAGACGATAGAATACGGCTGGTACGGCGCGAGAAACCTCCTCGCCCCATTCCAAGGACGGGTCTGGAATATCATAACGCCCGTGAGCCCTCAGACGATCGAGATCCCTGTCGCAATCGACTTCGAAGCTCTGAAGCGGGACGCCCTCAATGACGTCGGGAAATACGGTCCGTTCTTCAACTGCCAATCCCAGCTGCTGACCAAGCTAGGGAACAATGCCCCAGCAGTGACGATCCTGGTGTTGAGCCTCGTCTATGCAGGCGCATTGGTCTTTGCGCCGTGGTTGCTCATCGTCCTGAGTCATCACCTTGGTATCAAGATTTGTGGTCGCAAGCCCATCGATCTGATTCGYCTGGGAGACACCGAAGCGGACGAGATTCCGTGGTTGCTGGCTGAGACGTTCGACGACGAAGATGACTTGCCAGCTGACGTTTCTCCCGTGGACGATGTCCCTGCCGAAGCCAGTCGTGACTCATTCCTTGACCTCACCGACGAAGACCAAGCCCTCGCTGCGCTGCAATACGTGTTCTCGGAACTGCCAGAAGACGAAGTGACCCCTGACGCTCTCATCCTTGACGAGGCCGTTCAAGCCTGGAAAGCCATGGTCGATGTGCAAGAAGACCAGCCTCTAGCCGAGTACTACGAGCCTGTCGAAGGAGACTCACTGACCTCATGGGCAGAGGACTTCGTTCGATGGGCTCTCCAGGAGATCCGCACGATCGGGTCGGCGGCTCCCATCGCCCGTGATTTGGTTCGATTCCTCTTCGCGCTAAAGGACAACGTCGTTTGCTTCGCGGAACCAATGCTCCAAATCTTGAGAGTGGCTTTCAAAGCCATCGTTGAGCTCACCCGTCAGTTGGCTCCAGAGCTGGTTCGTCTCGCGTCACGATTGGTGGACCTTGCTTTCGGACACCAAGTCACGAGGAAACTGAAGGCTGCCTGGTTTGGGGCAGAGCTCCTCAAAGGGCACAAACTGAGCGTCCAACATCGGATTCGCGAACAGCTCGCCTTCTCACACTTCTTGGCCGAGGGAAACTTCGATGACCAATACAAGAAGGACACAGCTGAGCTACGGGCGGCCTACAGAGCCAGTGATGTTGACTGGGACAGGCTCCAAGCCATGTCCGAGGCGGAATTGGGTTCAAAGAAGCGTTTCGTCCATCGAGGTCCTCGTGGTTTCGGAGGTCCTGTCTGGAAGCCATTGCCGCTGCCACGATCTGCAGTCATGTCAGAGCAGGAGCTCGAACTCCTTATGGCCGAAGTTGAAAAAGAGAACAAGGCCGGAAATCCTGTCAACTTCAATGCAAGAGTTGACCAATACTTCACCGCGCGAGTAGCGCGATTGACTTCTGGCGGGACAGACCTCGCCACAGATGGCGCCCTTTTGGCAGCCATTCGCCCAGCGCAAGCCGAAGCTTCGCTGATCCGTTATACCTACCACGGGATCGAGCCAAATACAGCTCTAGGTACTAACACCCAACCGATGACACCCGAGCGGCGAGACCGACTCTTGGCGATTGCTGATGCCTACTACGAGACCAACAAGGATCTATTTGACGACCCCAAGTTGACGCCTCCTGAGGCCACCATCGAATGGTGGAAGCACCGCGGCATGACCAAATTCAACACGACCGCGCCTCTCAACATGGCCTCCAGGGCTCATGCCATCGTGGACGGGCAAATGACCGCCATTGTTCGGAACGTATACGAGAAGCTCAAAGCCGGTGAGTATCCTCACCAATACTACGCTGCGAAAGTCAAACAGCAAGCCGTCCCGGCTCGAAAACTGGTCACCCCTGACGCCAATGGAGACCTCAAGCCTGTCAGGACCTTCGTCGCGCAAGACCATCGTTCAACGGCGGCTGACTGGACCGTGGGTCTGGAACTGAAGAACCGTCTGCCTGGCGAGAACTCAGGCGAGTCATCCAAGATGGCCGCCGGCCAAGGTTACTCCCCTCTTTTCCGGAAAATCCGCGGAAAGGAGAACATTTTCATGGGAGACATGGCTCGCTATGACAGCCAACTCGAGAGAGATCATTTCTTCATGCTGGACCGTTGCTTAGAACGAGGCGTCTCTGACAAGGTTGTTCGGTCAATCCTCCAGGCCAAGCACGCTGCTATGCAATCATCTTACATTGCAGTGCTTTCTCTGCCTCGAGACAAGCCACTCGAACCTTTCCTCGCTCACGCGCGAGCATCGATGAGAGATTCCACAAAGGCTGCAGTGGGGTACGAAAATTGGATCCTCAAGATCCGATCCGGGGCCACCGGAGAGTCTTCGACCAACTGGACCGACTCGAAGACCTTCCGCTTGACGTTCGGACTGATCGTG

> Contig 2

TTGCGTGAAGGACAGCGCGATGCTCTTGAAACTGCCTTGAGACCACAAGTGCGTATACTGACATTTTCGGATTGGTATGCCGACCGCATGGCTTGGGCCGCTTCGGGAGGCGCCCCTGGAGCCAAAGTCCAGTGGGAGCCTCAAGCCAAGGCCGACAGATTGAATAAACGAGGTGCGTTGCTATTAATTCCAGAGAGCCACATACTAAATATCCTACGCCAAACGTTAAAGCCTTCACTATTTAGCAAAGCAGCCAAGAAATTCGAGAACGGTAAAATACGTAGCATTTGGAATACTAGTATATCGTTGTATGTTATCCAAGCTTACGTATTACATCATTTCGAGCGCGCTTGCGCACCCGGTACTTGGAATACATCGGCAGATAACACGTTCTCAAAGATGAGGGGAGACATCAAACGTTTGCTTGGTCTAAGCAAGCATGGCAACCATGGGTTGATGTGGGATTATTCTGACTTCAACATCAATCACACAGCTGAGTCGATGGTCTCGCTCTGGGAAGAGACGTTGAACGTTATGCTACCGAGGCTTGTCGCCGACACACCTGGTGAACTTGAACAATGTCGTTTGGACTTAGAACTCTGCGTCGCGTATATCGTGAATGCCAAGAGCAACACTTTCTTGTATAACCCGGAAACAGAATTTGGCAGCTATGTTTGTCGCAGTCTACAGAGTGGGGAACGCGCAACTTCTTTCGTTAATACTTTCAATTCACGCGCTTATGCTTACATACACGATCGGGTATCAATGCGGTTATTCAATAGGACACTATTACTTCCACAACTAGCTGCGCATCAGGGAGATGACGTCTTCAGAATCGTGGCTTCAATTAGTGATGGTGTCCTGGCGTGTGCCTTATTTAATTTGTTGGGATATGCTGGGCAAGTATTTAAGATCATGTTAGAATACAACCCTCGCGGTGAATTCCTGCGCCTCCACTACGACGGAGTAGCCAACATAATCGCTGGTTACCCAGTTAGATCGTACATGGGTTTAATAACTGGAGAGTTTTTCCGCGAGAACGTCATCGATCCAGGTGACCGTGCCATGGCATTTTGCGACCAGTTTGGGAAAGTACTTCGCCGTGGGGCAGTGTTGCCACAGCGAGTGTTAGATGTATTGCTCCGCGATCACACCGCCTTAACTTATACTCATGATGGAGTCCAAAGACGTGTTACACCTGATTTGACGATGTTGTTCACTCCTTCAGCACTTGGGGGATACGGAGCTAACGCTATTACAGCGCAAGGCACGGTCGCACTAGGTAGCAGCCTCCAAGCCGTACCAGATGTTTCGGGCGACGCTTTGCAACGAGAGACGGCTAGTGTTGGTTACGCGATAGCTATACCGTCGGGTCATGGCAAAACCACTCTCAAGAATAGGTACCCTGAACTGTTTTACGATCAAGATGATTTTACTAACCAACCCAAGGTTGCTAGTTTGATTGCTGATGCTAAACGCAGGGCCAGAGGCGGTGATGCCGGGGGATGGGAAGATTTAGACGCCTATCATAGAACAGCCTCCATCCCTCGTGACAAGGTATTGTTAACCTGGCATGCTGGGACCGTTCCTACCGGTTTCCGTTTGATTGCAGCGTTAATGGTGGACGCCAACCGCGAGGGAGTCGTCTTAAAACGTACTGATGAATATTTCTTCGTTAACATACACCGCCACTCAACATTGAGTATTAATTTGTTTAGCAACCATAATGAATTGGCTAACGAAGCTGTTCGCCTTGCCAAGATCGCCAAGAACAAGGAGTTTGTTCCAGGTTACTCAGTCAGGAGATTCTTAACTTATAACACTCGAGTGAAGGAGCGTCCGGAATACCACTTCCCACCGGTACCTGTTCGCGCCTTTTACGCCGGCACTGCTGGACAGATACCTGATGTAGGAACAGCGATCAGGTATCAACTCGATTTTAGTACTGTGACCAGTATTAAGAGGAGTATTCTTAATTCAGCCTTACCAGGTGCTTATCCAGCTGCCAAGCTGTCTAACACTCTGGCTAAATACGCTAGTGAGTTAGATAAGTGGATGCGGATTAATAAGCCTAGTACCACTATTCATGTTACAGCAATGCCACCTCAACAGGTTTTAGATGAATTCAGCTCCTATGCTGCTATGTTCACTGGAAGTTTATATAGTTTCCAAGGTGATGTTTCTACAGCACAAGGCTTTGCACATGCTGTTAATCTGTATTTCTCCACAATCGCTGACGATACCAATATTTCGTCGGCATATTTATTTGCGCGCCCCAACTACGGTGCATTCCAACATCTTGTCCGGCCAACTGGATGCTCGTCATTCGAGACTCTGGTCAACATTGTTGATGCATCCCCTCTCTTAGCATCTGATACTGGACATGCTGGGAGGTGGCTCAATTTAATTAACTCAACGACGCGCGCCAAAGAATCGAGGGTAGGTATCAGTTATGCTAAATTACATAAACGGATATCGGCTTCAACGTTTACCGCTGAAGAGAAAAGTAATGCTTACAACTTCCTTGTAGATTACTTTAGAGGCAACTTGAGTCTCTACCCACCAACAAAGCGAGTATTATCTCCAGTAATAATCTCACTATCAAGAATTTGTACGCTAATGTTTGTTGAATCGAACTTCATGTACTTTGCACGATTAGATATAATCACTAGATATGATTACCTATGTGTGTTAGAGGAACATGCGATCAGTAGATCTCAACAGCGCTTGTCTAGTATACCTAGTCAAGTTCATAGCACCCCTATTCAGTACATTGATTAG

> Contig 3

TGCTGCAACAATTTTCAACACACTGGACTTACCAGTGTGAACCTATAAAAGGCTGGGGATTCCTTAATCCAGAACCGTCCAGAGAACTTTTCGTTTGAGGTACGAAAGACGATGCGAGCCGAAAGCGTCACTTAGAACCTTGTCCGTTAGATTGATTTTGATAACCCAGAAAAAACGTGCCACGGGTAACAACACGCTAAACAACTGGAATCTGCTTTACAGTCAAAGCAGCAAAGACCAGCGACCTAACACCTTTAAGAAAGACCGACTTGTGCTGGGGAACCGCCTACTCAGCTTTTCACCGGAACCCGAAGCCTTAGCGATTTTACGAATCGAACGCGCTACTGAACGATAGTGCTCTTTTCCAACTGCGTATGAGCATAATGACGCATGCCCCAAAGCGTCAATCTCGTATCGAAACAAATGACTCGAGACGGGTTATCACCAAAGTGACACTACCACTTTTGCCTTAGATGTCCCAACAGAGAAAGATAGCGTGTAAGGGGCGGCTACCACCACAGGATATAGCAGCCGCAGGTGTTCGTCGCCGGGAGACGCGCCTTGCTGAGGACCGAACGACGAAATAAAGTAGGACCTGACTCGTCTCGCAATGACTGTTTTCAGATGCCGTGGACTTTTGACGAGTCTCGCGCGTATGGTCCGAACGCGGGGATCGAAAGGAGCCACACGTATCTTGCATTGGTCTTACGCATACACGTAATACTACCATCTATTGGTCTTACGCATGGAGATCACACGTAACACCGCATGTTGCCGAGGCGTAACTCTCGGTGCAAGGCGTGGGTGAGTGGCCGGCCGAAGCCTCATCAGTCTCGCTGCGCTAAGGCAAACGACGGACTGGTGTGAGAAAGGGAGGAGAGAGGGATCCTGAGTAACGACAGACTACGGTAGGAAGGAATGTAAGGGTGGGGCTTAGGAACGAATCGAAAGCCCGCGGTGTGGAAACTTGGAGGAAAGAATAGGACTTAGGACGAATCGAAAGCCCAGGGTGTGGAGTTACTCACTACCTCCCCGCTACTACACCGCCAAGGATGTTGGATCGTTCCTGCCTCAGGCAAGGATCGGTATGGCATCTCCTAGCGGTCGCGGTACATCGGTCAGAGCATGGCTCGTCTCGGATACCTCCGTTCTCGTCGCTACTTACGGACCCGAAGGCAGTAATCCCGAATTCCGGAATTACGTCGGAGGGATCGGTGGTAAGTGCGAGACTGGAGAATCCTTAACGGATGCTCTCGTCCGCGAAGTCAAGGAAGAGGTCGGAATTGACGTCTCATCATGCAATGTTGAGATATCGTCAGTCGAATCTCATCCCGATTTCACCGTCGTCTGGGCCATTGTGTACTGCGGAGAAGAGAAAGATATCTCAGTTCCCGAGTCAGAGAAGTCGAAAGTAGTCGACCCTCACTGGGTTCCTTTCTCAGAGATATCAGCACTCGAAGTTCCTGGCATCATGCATCCTGATACGTTGACAGCCTTCCAGGCTGCTAAACGTCTCTTTATGCAGGGTGCGTTGTACACTGCGACACGTGGTTCAGTGCCTTCGGCAACCTGGTTGCGCCGTGACGACCCGGATCAACTACCTGGCCCCGAGTGGAACCTGATCATGATTAACAACAAGGATGGCCTCTGTGTACCATACGCTATCCAAGCTGTGTCGGGTCAAACGTTCGACTTCAACGCGTTGGCAGCTGCTTTGCCTGATGCCGGATACGACTACCGTACACTCGTCGACGACCAAAGCCTTTGGGCAATGTGGTCTGCTGACGAACGTGCGTGGGTTGTCGGTAACCCGACGACGGCAAAATATTTCCTGTACCACAAGCGCAATGGCCATACTGCTCACGTAGATGGTTTGATACGCAGTACGGACCCCGCACAACAGTACGACCGCGTGTTCTATCCACAGGGCCGCAACTCTGAATTCTACCCTGTCCTCGAAGAGTTTAATGCATACGCGATGAGCGCAATCAAGGGTCTCACCGACCCAGGCGACCGTCAGCGTATCTACGGACAAGAGGTTGAAAAACTAATGGCCATGCCTGACCAAGACCGTCACAACTGGTTGGAGCGATGGTACAAAGTTGCCGTTCCTAGTGACTCAGCCGAACACACACCGGAACCTGTGCCTGCCACTTCTGTCCAGCAGCCAGCCGAGATTAATGGAGTCACCTCGCCACCTCAACAGTCCCAAGCTGCTCCTAGTTTCGGACTTACACTAACGCAGCCGGTCGTCTCCAGCCTTAACTATTCGGGTATGGCTGCACAAATAGGTAATGACGGCCCTCTGCGCCTCACAGGTTCATTGGACACCGTGGCAATTACTGCCGACCGTCGTGCCCAGGTCATTGATATAAATGACCCTGAGCTTATATTGCGGGCACCTGAGATTCAGCATGCCGTGTCCTTTGCAATCCCAGGAGAATAAGTG
